# Coexistence of antithrombin deficiency and suspected inferior vena cava atresia in an adolescent and his mother – case report and clinical implications

**DOI:** 10.1186/s12959-021-00360-0

**Published:** 2021-12-22

**Authors:** M. Müller-Knapp, C. F. Classen, R. Knöfler, C. Spang, C. Hauenstein, T. Heinrich, F. L. P. Gabriel, J. Däbritz, D. A. Reuter, J. Ehler

**Affiliations:** 1 Department of Pediatrics, Interdisciplinary Pediatric Intensive Care Medicine, University Medical Center Rostock, Rostock, Germany; 2 Department of Pediatrics, Oncology and Hematology Unit, University Medical Center Rostock, Rostock, Germany; 3 Department of Pediatrics, Division of Pediatric Hemostaseology, University Hospital Carl Gustav Carus, Technical University Dresden, Dresden, Germany; 4 Department of Anesthesiology and Intensive Care Medicine, Interdisciplinary Pediatric Intensive Care Medicine, University Medical Center Rostock, Rostock, Germany; 5 Institute of Diagnostic and Interventional Radiology, Paediatric Radiology and Neuroradiology, University Medical Center Rostock, Rostock, Germany; 6 MVZ für Humangenetik und Molekularpathologie GmbH, Rostock, Germany; 7 Institute for Clinical Chemistry and Laboratory Medicine, University Medical Center Rostock, Rostock, Germany; 8 Department of Pediatrics, University Medical Center Rostock, Rostock, Germany

**Keywords:** Pediatric hematology, Pediatric thrombophilia, Antithrombin deficiency, Inferior vena cava atresia, Thrombosis, Genetic analysis, Gene mutation

## Abstract

**Background:**

Antithrombin deficiency (ATD) is an autosomal dominant thrombophilia presenting with varying phenotypes. In pediatric patients with ATD, thrombosis typically develops during the neonatal period or adolescence. However, to date there are no consistent recommendations on the therapeutic management of children with ATD. Inferior vena cava atresia (IVCA) belongs to a range of congenital or acquired vena cava malformations and is described as an independent risk factor for thrombosis.

The present case report explores two cases of combined ATD and IVCA in an adolescent and his mother.

**Case presentation:**

A 14-year-old male presented with extensive deep venous thromboses (DVTs) of both lower extremities as well as an IVCA. The patient had previously been diagnosed with an asymptomatic ATD without therapeutic consequences at that time. His mother was suffering from an ATD and had herself just been diagnosed with IVCA, too.

The DVTs in the adolescent were treated by systemic anticoagulation and catheter-directed local thrombolysis causing favourable results. Yet, despite adequate oral anticoagulation the DVTs in both lower extremities reoccurred within 1 week after the patient was discharged from hospital. This time, thrombolysis could not be fully achieved. Surprisingly, probing and stenting of the IVCA was achieved, indicating an acquired IVCA which could have occurred after undetected thrombosis in early childhood.

Genetic analyses showed the same mutation causing ATD in both son and mother**:** heterozygote missense mutation c.248 T > C, p.(Leu83Pro), within the heparin binding domain of antithrombin. This mutation was never reported in mutation databases before.

**Conclusions:**

To our knowledge this is the first case report discussing combined ATD and IVCA in two family members. Since ATDs present with clinical heterogeneity, taking a thorough family history is crucial for the anticipation of possible complications in affected children and decisions on targeted diagnostics and therapeutic interventions. Affected families must be educated on risk factors and clinical signs of thrombosis and need an immediate diagnostic workup in case of clinical symptoms. IVCA in patients with ATD could occur due to thrombotic occlusion at a very early age. Therefore, in case of family members with IVCA and ATD ultrasound screening in newborns should be considered.

## Background

Antithrombin deficiency (ATD) is an autosomal dominant thrombophilia caused by mutations in the *SERPINC1* gene [1, 2]. ATD has a prevalence of 1:2000–5000 [1, 2] and more than 300 different defects with varying phenotypes are currently listed in common mutation databases (ClinVar, HGMD, gnomAD). There are two different types of ATD: patients with quantitative type 1 ATD generally have a higher risk for thrombosis than patients with qualitative type 2 ATD [2, 3]. In pediatric patients with ATD, thrombosis typically develops during the neonatal period or adolescence. Thrombosis tends to be provoked by additional risk factors such as immobilization, and is associated with severe morbidity and mortality [4, 5]. Inferior vena cava atresia (IVCA) belongs to a range of congenital or acquired vena cava malformations and has been observed as a risk factor for thrombosis [6, 7].

Here, we explore two cases of combined ATD and IVCA in an adolescent and his mother and report on new etiologic and therapeutic perspectives concerning this relevant condition.

## Case presentation

A 14-year-old male adolescent presented to the emergency department with subfebrile temperatures for 1 week and localized pain in his right popliteal fossa for 3 days. Prior to the onset of these symptoms, he had been immobilized for several days following a minor sports injury.

The adolescent had no permanent medication and no prior medical history except for an asymptomatic ATD diagnosed at the age of six by functional antithrombin assay (antithrombin activity of 57%, age adapted reference: 77–125%). Since at that time, there were no clinical signs of thrombosis, the diagnosis of ATD did not lead to any therapeutic consequences. Screening for ATD at this early age had been carried out on parental request, as his mother was diagnosed with ATD in her early adulthood. Interestingly, his mother now reported that she had very recently been diagnosed with IVCA (preexisting chromogenic test results showed an antithrombin activity of 50% for the mother, the age adjusted reference range being 80 to 130%).

The coexistence of other hereditary thrombophilic disorders in our patient and his mother (protein S deficiency, protein C deficiency, factor V Leiden mutation, prothrombin-mutation, antiphospholipid syndrome) was ruled out by respective laboratory analyses.

Laboratory blood analysis in the emergency department showed markedly elevated D-dimers of 25 mg/l FEU (reference: < 0.5 mg/l FEU) and of C-reactive protein (CrP) of 184 mg/l (reference < 5 mg/l). Antithrombin activity on admission was reduced to 61% (age adjusted reference 83–118%).

A vascular ultrasound examination upon admission confirmed the clinically suspected thrombosis of the right lower extremity involving the external iliac, common and superficial femoral as well as the popliteal vein. The ultrasound examination of the left lower extremity veins did not give evidence of thromboses upon admission. The patient was treated with continuous infusion of unfractionated heparin at a therapeutical dose including several bolus administrations and antithrombin (4000 IE within the first 36 h) was substituted reaching levels in the target range between 80 and 100%. Subsequent monitoring of the activated partial thromboplastin time (aPTT) showed an insufficient response to heparin treatment (aPTT was 29 s before the administration of heparin and did not exceed a maximum value of 33.6 s during heparin treatment). The anticoagulative therapy was therefore switched to direct thrombin inhibition by argatroban at a dose of 1.5–2.0 μg/kg/min, which led to target aPTT values of 50–60s.

Due to the elevated CrP levels and subfebrile temperatures, empirical antibiotic treatment with intravenous cefuroxim was initiated.

**Fig. 1:**
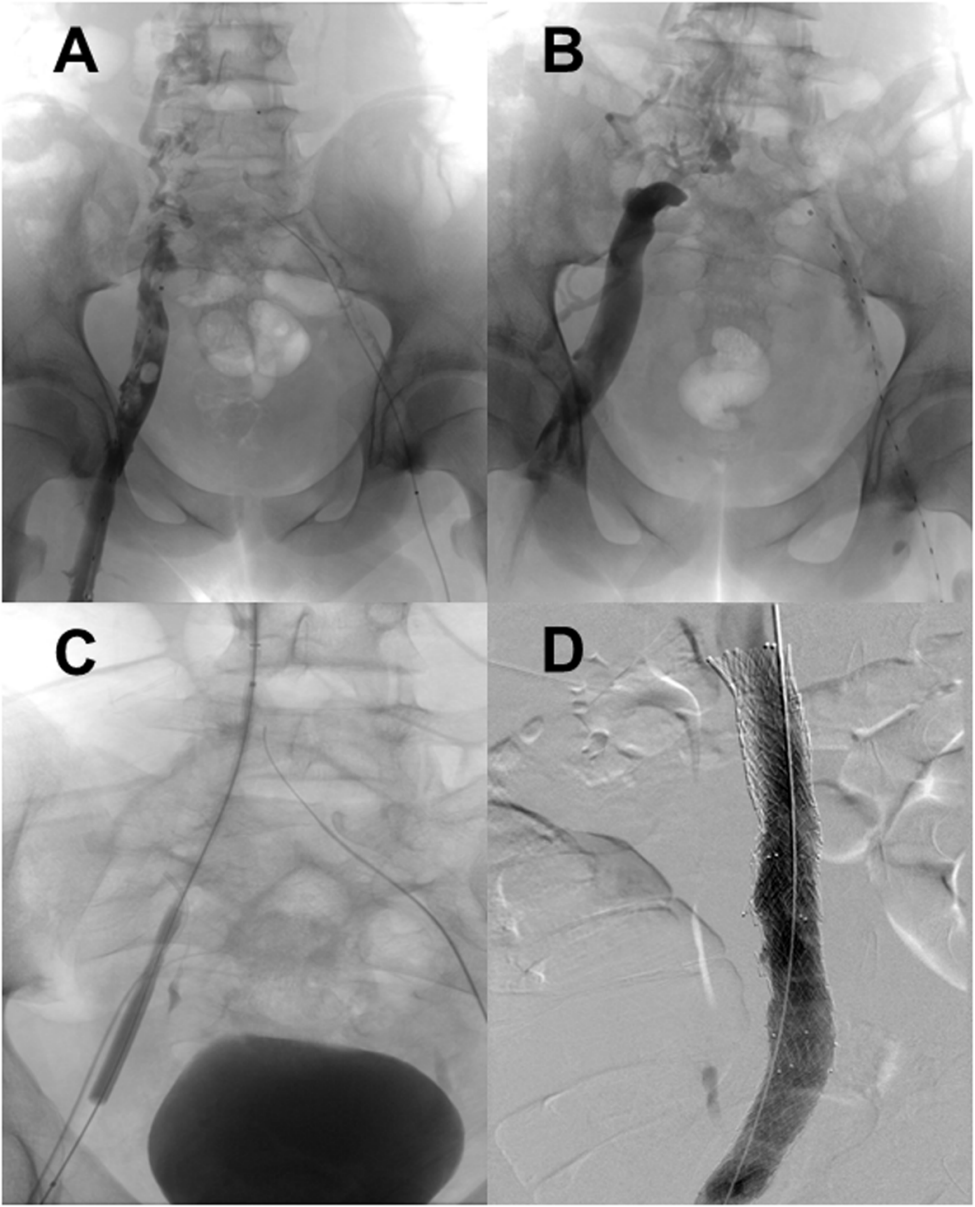
Angiography images in our 14-year-old patient. **A** Angiography shows a thrombotic right pelvic axis with suspected inferior vena cava atresia and prominent paravertebral collaterals; **B** Angiography confirms inferior vena cava atresia; **C** Location of the occluded inferior vena cava by rendezvous-technique between superior and inferior vena cava; **D** Recanalized inferior vena cava after successful stenting

A magnetic resonance imaging (MRI) scan on day three confirmed a complete DVT of the right lower extremity, and now additionally extended thromboses of the left lower extremity and thrombosis of both iliac vessels. Furthermore, it revealed an occlusion of the inferior vena cava (IVC) with prominent venous lumbar collaterals, suggestive of IVCA. Surrounding the lumbar vertebrae, several of these collaterals appeared to be fully thrombotic, which was confirmed by angiography (Fig. 1A).

The following day, a catheter-directed local thrombolysis using recombinant tissue plasminogen activator (rt-PA) at a daily dose of 0.25 mg/kg was initiated in both extremities and carried out for a total of 48 h. For the right leg, an additional ultrasound-accelerated thrombolysis using the EKOS® system was performed.

During hospitalization, the anticoagulative therapy was switched from argatroban to the vitamin K antagonist phenprocoumon with a target International Normalized Ratio (INR) of 2.0–2.5. Oral anticoagulation will most likely be continued lifelong due to the combination of extended multilocal thromboses with underlying ATD, as well as IVCA, which in itself is a risk factor for thrombosis.

The patient was discharged on day eight after hospital admission. An ultrasound examination upon discharge showed full recanalization of the femoral and iliac veins of both legs.

During a routine follow-up 1 week later, the patient presented free of any clinical complaints. However, despite continued oral anticoagulation since hospital discharge, vascular ultrasound now revealed extended re-thrombosis of both the right and left pelvic axis and the right lower extremity, thus leading to a second hospital admission and re-induction of therapy with both intravenous antithrombin and catheter-directed thrombolysis.

Recanalization of the right lower extremity and pelvic axis was subsequently achieved, whereby IVC occlusion was confirmed (Fig. 1B). However, the left pelvic vessels remained thrombotic even after 5 days of catheter-directed thrombolysis.

Surprisingly, one of the angiographies allowed for location of the occluded area of the IVC by rendezvous-technique between the superior and inferior vena cava, which prior to this had been thought to be atretic (Fig. 1C). The vessel was successfully recanalized using balloon dilation and subsequent stenting (Fig. 1D).

Throughout the clinical stay, the patient’s oral anticoagulation with phenprocoumon was intensified to a new target INR of 2.5–3.0 in order to prevent re-thrombosis.

The patient was discharged from hospital on day seven after admission. Regular follow-up examinations at our outpatient clinic did not give evidence for any new thrombotic events to date (time since first occurrence of thrombosis was 9 months).

Concerning the thrombotic left iliac vessels, a follow-up angiographic intervention relying on special equipment was performed, but recanalization could not be achieved.

Fortunately, the IVC of the patient’s mother could also be recanalized by angiographic intervention.

## Discussion and conclusions

In pediatric patients ATD increases the risk for thrombotic events by factor 300, and morbidity and mortality of these thromboses tend to be high [4, 5, 8]. Yet, to date there is a lack of consistent recommendations for the therapeutic management of children affected by ATD [2, 9].

Here, wepresent an impressive case of hereditary ATD in a patient who did not only suffer from extensive thromboses, but also presented with IVCA, which in itself is a known risk factor for thrombosis [6, 7].

During the course of treatment, angiographic recanalization of the patient’s IVC was achieved, thus pointing towards an acquired and not congenital IVCA. Retrospectively, an obliteration of the IVC, maybe at an early age and most likely due to thrombosis, has to be discussed. This is supported by the presence of extensive lumbar collaterals which were thrombotic upon admission, thus possibly promoting the recent development of thromboses in the patient’s lower extremities.

On this issue, Mabud et al. recently described an association between hereditary thrombophilias and IVCAs, hypothesizing that in many of these combined cases, IVCAs might not represent primary agenesis, but instead arise secondarily due to thrombotic events [10]. This is consistent with previous case reports referring to the rare KILT (kidney and inferior vena cava anomaly with leg thrombosis) syndrome [6]. Most importantly, a recent and large study by de la Morena-Barrio et al. found a high penetrance of IVCAs in patients with the homozygous antithrombin Budapest 3 variant, a form of ATD with generally high risk for thrombosis [11]. To our knowledge, this is the first study showing an association between a specific form of ATD and IVCA.

Thrombotic events that could cause IVCA are most likely to develop during early childhood or the neonatal period, when the risk of thrombosis in patients with ATD is known to be particularly high. These early thrombotic events resulting in subsequent IVCA might not present with typical clinical signs such as bilateral leg swelling due to the relative immobility of the young patients. Thrombosis may therefore be undetected and untreated [10].

During the clinical stay of our patient, intensified reviewing of the family history revealed that the mother of our patient had herself been diagnosed with ATD at the age of 24 years and had suffered multiple extensive thromboses from the age of 12 years onwards. Additionally, she was diagnosed with IVCA just 5 months prior to her son and was awaiting angiography herself.

Genetic analysis in our patient confirmed the same mutation in the *SERPINC1*-gene that had already been detected in his mother (heterozygous missense mutation c.248 T > C, p.(Leu83Pro), within the heparin binding domain of antithrombin; reference transcript: NM_000488.3). To date, this variant has not been reported in mutation databases (ClinVar, HGMD, gnomAD). Following guidelines of the American College of Medical Genetics and Genomics [12] and considering (a) in silico predictions of pathogenicity, (b) functional consequences (antithrombin assay), and (c) familial segregation the variant was classified as “likely pathogenic” (class 4 mutation). One might speculate that initially insufficient therapeutic response to heparin in our patient was influenced by this particular mutation type affecting the heparin binding site.

As mentioned above, antithrombin activity had been reduced to 61% on admission (analysis performed with the INNOVANCE® Anti-Xa assay, age adapted reference 83–118%). To further classify the ATD, a blood sample was sent off for combined analyses of functional antithrombin assay and antithrombin antigen. This sample revealed an antithrombin activity of 40% and antithrombin antigen of 33% (quantified by radial immunodiffusion, age adjusted cutoff 77–125%) thus indicating a type 1 ATD. Concerning the difference in antithrombin activity between the different samples (61% upon admission and 40% during repeat analysis) it is known that antithrombin analyses can be influenced by various factors such as pre-analytical variables or circadian variations [13]. Most importantly antithrombin measurements are not reliable in the acute phase of a thrombotic event and should thus be re-evaluated 3 months after the event (which holds true for the second measurement of antithrombin activity of 40%) [14].

Regarding the preexisting laboratory test results, the mother had significantly reduced antithrombin activity of 50% (via chromogenic assay, age adjusted cutoff 80–130%), too. Antithrombin antigen values were not available.

It is known that hereditary ATDs present with great clinical heterogeneity [1]. Mutations affecting the heparin binding site are generally classified as type II mutations and expected to be associated with medium thrombogenic risk [1], except for antithrombin Budapest 3 *homo*zygotes, where the risk for thrombosis is extremely high. However, this particular mutation shows reduced antithrombin functional activity and reduced antigen levels and is therefore characterized as type I mutation. The substitution of leucine by proline at position 83 affects a relatively conserved residue in the serpin superfamily. This change probably disturbs the folding of the native conformation of antithrombin (due to the conformational rigidity exerted by the proline ring structure) thereby leading to the type I deficiency.

In our adolescent patient, the mutation led to multiple extensive thromboses at the age of 14, which occurred after a short period of immobilization. He later developed re-thrombosis despite adequate anticoagulation and without further risk factors present. We speculate that he possibly suffered from an undetected thrombosis at an early age, resulting in a secondary obliteration of the IVC. Notably, the family history finally revealed a strikingly similar clinical course in his mother, which led us to speculate that she too might have suffered from an undetected early childhood thrombosis leading to obliteration of the IVC.

It has been observed previously that clinical severity for antithrombin heparin binding site mutations significantly depends on the individual pathogenic variant [15] illustrating the complex genotype-phenotype relationship of *SERPINC1* genetic alterations. The missense mutation c.248 T > C, p.(Leu83Pro) identified in the two members of our family (mother and son) was detected in heterozygote state. It is known that homozygosity for *SERPINC1* mutations in type I deficiency and in most type II deficiencies is incompatible with life, whereas homozygosity for specific type II mutations, namely AT Budapest 3 (p.Leu131Phe), is often found in the background of pediatric thrombosis cases, due to the high frequency of this mutation and due to its very severe behavior [16].

To date, there are no guidelines for the therapeutic management of pediatric patients with ATD [2, 9]. However, after carefully reviewing both our clinical case and the related literature, we would like to propose the following cornerstones for the care of (potentially) affected children:

1. Family history as a base for diagnostic deliberations
  ATDs present with great clinical heterogeneity, thus conducting a thorough family history is crucial for anticipation of possible complications in affected children. In case of adults with early and extensive thromboses associated with a proven ATD, the indication for targeted thrombophilia diagnostics in their children should be considered and discussed on an individual basis with respect to potential legal restrictions regarding testing of asymptomatic minors.
2. Education of affected families and regular clinical monitoring
  In case of positive ATD diagnostics in a child, the family should be advised about risk factors for and clinical symptoms of thrombosis. Medical attention should be sought immediately if risk factors or symptoms become present. In case of high risk thrombophilic situations such as complete immobilization for several days after surgical procedures primary prophylactic anticoagulation should be performed.
  Thromboses in young children with ATD tend to occur in otherwise uncommon locations (e.g. the renal/ cerebral veins or upper extremities) [3] and this should be taken into account for any symptom constellation in the child.
3. Prophylactic and therapeutic anticoagulation
  Long-term prophylactic anticoagulation in children with asymptomatic ATD is generally not recommended but may be discussed in cases with extensive risk (e.g. uncommon homozygous ATD and elder sibling with catastrophic thrombosis at an early age) [17].
  After the first occurrence of thromboses, long-term and possibly lifelong anticoagulation has to be considered.
4. IVCA as an indicator for highly thrombogenic ATD
  To our knowledge, there are only two other case reports describing singular patients with ATD and IVCA to date. Both reports regard the occurrence of ATD and IVCA as unrelated events [7, 18]. However, recent data [10] as well as our clinical case with a suspected IVCA that could be recanalized support the hypothesis that IVCA in thrombophilic patients may not necessarily be due to primary agenesis but instead arise following thrombotic occlusion with subsequent obliteration at an early age.
  If parents or other family members with ATD are known to suffer from IVCA, ultrasound screening of newborns should be considered immediately after birth and – in case of asymptomatic ATD – possibly be repeated until the child is fully mobile. This could help to diagnose asymptomatic thromboses of the IVC and enable early treatment in order to prevent significant complications.
  In conclusion, the diagnostic and therapeutic management of pediatric patients with ATD should encompass a multidimensional and multidisciplinary approach.
  According to recent publications and our clinical experience, IVCA in patients with ATD should also strongly be considered as a possible residue of a previous thrombotic event.
  The specialized diagnostic and therapeutic management including decisions on possible long-term anticoagulation in children should be performed in experienced pediatric centers.

## Data Availability

The datasets analysed during the current study are not publicly available due to individual privacy matters but are available from the corresponding author on reasonable request.

## Abbreviations

aPTT: Activated partial thromboplastin time
ATD: Antithrombin deficiency
CrP: C-reactive protein
DVT: Deep venous thrombosis
INR: International Normalized Ratio
IVC: Inferior vena cava
IVCA: Inferior vena cava atresia
MRI: Magnetic resonance imaging
rt-PA: Recombinant tissue plasminogen activator
